# AI-Enabled Misconduct During Interviews and Its Possible Impact on Future Physicians’ Critical Thinking

**DOI:** 10.7759/cureus.114239

**Published:** 2026-08-09

**Authors:** Thomas J Papadimos, Stanislaw P Stawicki

**Affiliations:** 1 Anesthesiology, Surgery, and Critical Care, The University of Toledo College of Medicine and Life Sciences, Toledo, USA; 2 Surgery, St. Luke's University Health Network, Temple University, Bethlehem, USA

**Keywords:** faculty, medical resident education, medical specialisations training, residency interview, virtual interview

## Abstract

The competition for highly desired residency training positions in certain medical specialties has become intense. Annually in the US, there are multiple instances of an inability of the National Residency Match Program (NRMP) to accommodate each medical student’s desire for a seat in their residency of interest. Residency applicant interviews since the COVID-19 pandemic are virtual, initially for disease prevention, but now for cost savings (to both the institution and the applicants). This intense competition for positions has led to sophisticated measures by some applicants to manipulate the system by use of artificial intelligence robots (AI bots) during these virtual interviews. These AI bots gather information quickly in response to an interviewer’s question and allow the applicant to seamlessly respond. Here, there is not only a consideration of cheating during the interview, but a question of whether such behavior will affect the critical thinking abilities of future physicians. Academic misbehavior through use of AI during a residency interview may portend dishonesty in a successful applicant's future medical practice. We present this commentary as a cautionary tale to those involved in the selection process regarding residency training slots.

## Editorial

Critical thinking is being assaulted by the use of artificial intelligence (AI) in manuscript writing, in which the written word is not human-owned and attributed references are hallucinated by AI [1]. The same can be said of personal statements submitted for residency training. Of note, we may be entering an AI arms race in which applicants are implementing sophisticated methods to deceive residency directors during the interview process through contrivance of the spoken word [2-4].

The introduction of increasingly sophisticated chatbots predictably resulted in a corresponding surge in the usage of this technology by the public. When it comes to AI, the paradigm of “if you build it, they will come” seems to apply across societal domains [5]. However, in the process of widespread societal adoption and embrace of AI and its derivative products - broadly defined “generative” tools - an important component of what used to be inherently unique to humans seems to be undergoing atrophy [6,7].

We have reached the point where the use of echoborgs is coming into play for applicants. An echoborg “is composed of a human whose words, and potentially their actions, that are entirely or partially determined by a computer program" [8]. This work by Corti and Gillespie over 10 years ago demonstrated how it is possible to embody, or project machine intelligence through a human interface, and such acts can be readily identified [9].

We are entering a stage in human educational and scientific development where we fear and anticipate a loss of our critical thinking abilities, not simply because AI has arrived (and it is here to stay), but because those who will use it the most have not been groomed to use it as an adjunct, as contrasted to using it as a primary source for their thinking and analytic abilities. Using the above example of an echoborg: the computer will speak through the human interface, wherein the human is used by the machine versus the human using the machine.

Applicants are loading AI robots (AI bots for short) into their laptops, and make no mistake about it, these bots are powerful. They can amalgamate the entire history of the interviewer for the interviewee through Internet sources in seconds, if not microseconds. When the interviewer (that would be us) asks a question, the AI bot on the interviewee side of the screen can hear/decipher the question, search the interviewer’s publication history, work history, media platform opinions, and travel history, etc., and then deliver the most acceptable answer for that particular interrogative to the interviewer, either through an earpiece or on the screen. We are cautioning our colleagues to look for rapid left and right eye movements by the interviewee as they scan their computer screen, an inability to hold a gaze, and/or the presence of an earpiece. The interviewees can hang a "post-it" note or a phone on the screen, and these objects will be essentially transparent; the interviewer will see nothing. The competition for highly sought-after training programs, such as anesthesiology and orthopedic surgery, seems to call for desperate action on the part of some applicants.

The Turing Test was proposed by Alan Turing in 1950 [10], who was interested in measuring a machine’s ability to have behaviors that were identical to humans. His test was one in which he was seeking a machine’s ability to exhibit intelligent behavior. Professor Turing would not be surprised that we have reached the juncture where machines are able to tell a human interface what to say and do during an interview. This behavior or manipulation of the interview system is not surprising in view of what has happened since 2022 with ChatGPT (OpenAI), Claude (Anthropic), and Gemini (Google) [11-13].

Here we are addressing a form of cheating during residency interviews where a perfect storm of science, technology, and candidate desperation merge together in an event that can fool even the most able residency training program directors and interviewers. There are solutions that can be implemented; let us call them hack avoidance interventions. They may be expensive and may be deemed impractical by academic administrative bodies, and are as follow: In-person interviews, personal statements that must be hand-written (although we realize they can be copied from an AI source - maybe they should be done during an onsite interview), not allowing earpieces during virtual interviews, training courses for interviewers on how to spot AI bot hacks and how to conduct interviews, recruit the best candidates from your own institution, and employ your own institutional anti-hacking technology. There may be other interventions, but these are a starting point.

Serious concern arises among more mature educators and scientists regarding those who over-rely on AI during education and training in that they may have difficulty in embracing critical thinking in the coming years. Yes, we (the authors and their contemporaries) are older, more experienced, and more aware of the history and evolution of scientific philosophy of the 20th and early 21st centuries, but not so naïve as to think that any of this will go away. Nonetheless, we remain confident that, as we stand on the shoulders of our scientific and clinical forefathers, they too shall stand on our shoulders and find their way through a smaller, increasingly complex, and intertwined world.

Critical thinking fosters personal development, logical criticism, solid working relationships, and successful enterprises. Critical thinking propels the world forward. AI should not be a journey’s shortcut; it should be an adjunct to the journey. It should support critical thinking in solving problems at the edge of human understanding.
